# 4D Printing of Recyclable Lightweight Architectures Using High Recovery Stress Shape Memory Polymer

**DOI:** 10.1038/s41598-019-44110-9

**Published:** 2019-05-20

**Authors:** Ang Li, Adithya Challapalli, Guoqiang Li

**Affiliations:** Department of Mechanical & Industrial Engineering, Louisiana State University, Baton Rouge, Louisiana 70803 USA

**Keywords:** Mechanical engineering, Polymers, Mechanical properties

## Abstract

High-performance lightweight architectures, such as metallic microlattices with excellent mechanical properties have been 3D printed, but they do not possess shape memory effect (SME), limiting their usages for advanced engineering structures, such as serving as a core in multifunctional lightweight sandwich structures. 3D printable self-healing shape memory polymer (SMP) microlattices could be a solution. However, existing 3D printable thermoset SMPs are limited to either low strength, poor stress memory, or non-recyclability. To address this issue, a new thermoset polymer, integrated with high strength, high recovery stress, perfect shape recovery, good recyclability, and 3D printability using direct light printing, has been developed in this study. Lightweight microlattices with various unit cells and length scales were printed and tested. The results show that the cubic microlattice has mechanical strength comparable to or even greater than that of metallic microlattices, good SME, decent recovery stress, and recyclability, making it the first multifunctional lightweight architecture (MLA) for potential multifunctional lightweight load carrying structural applications.

## Introduction

Lightweight architectures and constructions have been used in almost all manmade engineering structures including aircraft, naval vessel, car, train, pressure vessel, pipe, bridge deck, offshore oil drilling platform, wind turbine blade, etc., due primarily to their high specific strength and stiffness, tailorability, and corrosion resistance. One example is sandwich structure^1–7^. In a sandwich structure, the core is the key to ensure lightweight and multifunctionality. Various types of lightweight core materials have been studied, such as foam core (polymeric foam, metallic foam, ceramic foam, balsa wood, syntactic foam, etc.)^8–10^, web core (lattice, truss, honeycomb, etc.)^11^, 3-D integrated core or foam filled web core^12,13^, laminated composite reinforced core^14^, grid-stiffened syntactic foam core, etc.^7,15^. Among them, foam cored sandwich has been popular. In sandwich structural applications, it is highly desired that foams have higher strength and stiffness, and at the same time, have ultralow density. Unfortunately, it has been widely accepted that the relative strength and stiffness are coupled with relative density for foams or cellular solids such as lattice structures^16–18^:

1 $$E/{E}_{s}\propto {(\rho /{\rho }_{s})}^{n};\,{\sigma }_{e}/{E}_{s}\propto {(\rho /{\rho }_{s})}^{n};\,{\sigma }_{p}/{\sigma }_{y}\propto {(\rho /{\rho }_{s})}^{n};$$

where *E* is the Young’s modulus of the foam, *E*_*s*_ is the Young’s modulus of the cell-wall material (solid), *ρ* is the density of the foam, *ρ*_*s*_ is the density of the cell-wall material (solid), *σ*_*e*_ is the elastic collapse stress of the foam (cell wall buckles), *σ*_*p*_ is the plastic collapse stress of the foam (cell wall yields), *σ*_*y*_ is the yield strength of the cell-wall material (solid), and *n* is the scaling factor. Based on the literature, *n* = 1.5 ~ 3, depending on if the cell is closed or open^16–18^. It is clear from Eq. 1 that, for ultralow density foam, the mechanical properties of the foam degrade significantly. For example, if the relative density is 10% and *n* = 3, the Young’s modulus and collapse stress become 0.1% of their original values. Therefore, the grand challenge in foam is how to achieve high strength and stiffness with minimal weight penalty.

In a certain sense, lattice structure is a type of open celled foam. By examining the formulation process by Gibson and Ashby^16^, it is seen that when they studied the open celled foam, they used a cubic unit cell or cubic lattice as the basic building block. When the foam is under uniaxial loading, each edge of the unit cell was treated as a beam subjected to a concentrated transverse load at the mid span. Clearly, the beam element can be easily bent by the concentrated transverse load, and with additional axial load transferred from the nodes of the unit cell to the beam, can easily buckle, leading to lower collapse strength of the foam. With 3D printing, we can join all the beams at the nodes of the unit cell, thereby eliminating the concentrated bending load at the beam mid span, and leading to increased local buckling load and higher collapse strength in the foam or lattice. Despite the excellent mechanical properties, current high-performance lightweight architectures cannot meet the stringent requirements for advanced engineering applications. For example, shape memory is desired for deployable structures in aerospace^19^, crack closing after impact damage^20^, etc. For another example, damage healing is required to extend the service life and recyclability is required for environmental protection^21,22^. Hence, development of multifunctional lightweight architectures (MLAs) with facile processability, good shape memory effect, decent recovery stress, damage healing, and recyclability at the end of its service life, is highly desired. Unfortunately, current 3D printable materials cannot satisfy the desired properties for MLAs.

Although various metallic microlattices with excellent mechanical strength have been 3D printed and shown as promising components for future engineering applications^23–26^, shape memory metallic microlattices have not been developed. Meanwhile, the manufacturing process of metallic microlattices via 3D printing is much slower, more energy consuming, more post-processing work, and limited to less available materials as compared to polymer 3D printing, impeding advanced applications of metallic microlattices as MLAs.

3D printable shape memory polymers is a promising solution to this problem. Compared to shape memory alloys, shape memory polymers usually exhibit better shape memory effect with lower price due to larger deformation upon programming. By applying shape memory polymers to 3D printing, the manufacturing of shape memory microlattices becomes possible. 3D printing of shape memory polymers is also known as 4D printing in terms of the additional dimension “time”, which imparts the printed objects with stimuli-responsive self-evolving features^27^. 4D printing exhibits a great potential to develop smart sensors, electronic devices, soft robots, biomedical devices, deployable structures, etc.^28–32^. However, 3D printed microlattices based on shape memory polymers for advanced structural applications have not been developed mainly because current 3D printable shape memory polymers do not possess the required mechanical strength and stiffness^33–35^. As a result, the 3D/4D printed objects are inadequate for load-bearing structures. Furthermore, insufficient shape recovery stress, a bottleneck for developing high-performance shape memory polymers, also restricts lightweight structural applications with 3D printable shape memory polymers. Most thermoset shape memory polymers have less than 2 MPa or even 1 MPa recovery stress in rubbery state^36^, which significantly limits the energy output during shape recovery. Currently only a few shape memory polymers with recovery stress larger than 10 MPa are developed, but none of them is 3D/4D printable^37–39^. Consequently, a breakthrough needs to be made to develop a high-performance shape memory polymer with high stress output for 3D printing so that MLAs can be obtained.

Pursuit of high resolution 3D printable high-performance shape memory polymers commonly means printing of thermosets, which can be fabricated through direct light printing (DLP) or stereolithography (SLA)-based technique. However, traditional thermosets cannot be remolded and have to be burnt or landfilled at the end of the service life, which results in negative environmental impact on potential structural applications. In order to improve sustainability of the MLAs, employment of vitrimer could be one solution^40^. Vitrimer originally represented a thermoset that behaves like a viscoelastic liquid at high temperature, and now it encompasses a broader meaning with thermosets composed of dynamic covalent bonds, which impart the crosslinked network with stimuli-responsive behaviors like controllable disintegration, self-healing property and reprocessability^40–44^. Likewise, most existing 3D printable vitrimers lack the mechanical strength and shape memory effect required for load carrying structures, such as space applications^45–47^.

**Figure 1:**
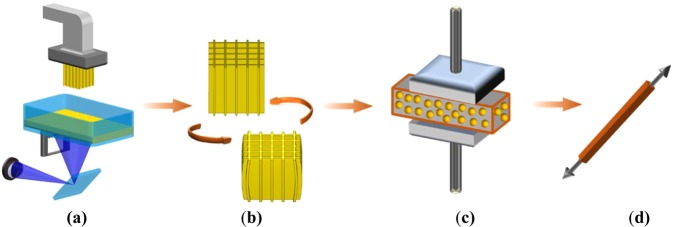
Schematic of recyclable and shape memory microlattices using 3D-RSMP. (**a**) 3D printing (direct light printing (DLP)) of advanced multifunctional microlattice structures using the 3D-RSMP. (**b**) Compression programming of the printed microlattice to a temporary shape and recovery to the original shape. (**c**) Recycling of the ball milled multifunctional microlattices under high pressure and high temperature. (**d**) The remolded specimen for mechanical tests.

To address the challenge, we first developed a new 3D printable and recyclable shape memory polymer (3D-RSMP) resin through integration of several desired features into one polymer: 3D printablility, high mechanical strength, good shape memory effect, high shape recovery stress, and recyclability. By applying 3D-RSMP to 3D printing, we further developed a new MLA with shape memory effect and recyclability for advanced structural applications by the following sequence: printing of 3D microlattices (Fig. 1a), investigating the effects of geometry and length-scale on the mechanical properties and shape memory effect of the microlattices (Fig. 1b), recycling of the microlattices (Fig. 1c), and assessing the recycling efficiency (Fig. 1d).

## Results

### 3D Printing of various geometries with 3D-RSMP

Bisphenol-A glycerolate diacrylate (BPAGA) or bisphenol-A glycerolate dimethacrylate (BPAGMA) have been widely used as a component in coatings, adhesives, and dental materials for decades, and lately they have also been adopted as a component in 3D printing resin formulation due to their photo-sensitivity and good thermal and mechanical properties. However, the multifunctionality of BPAGMA was not discovered until very recently^38^. It was shown that the UV-cured BPAGMA has good shape memory effect with high recovery stress and energy output. In contrast to BPAGA, the UV-cured BPAGMA has larger storage modulus in rubbery state, presumably attributed to the increased steric hindrance of the highly crosslinked methacrylate group^37^. The sterically hindered network allows for higher energy storage during the compression hot programming, and, thus, the UV-cured BPAGMA is able to produce large recovery stress (higher than 10 MPa)^38^. On the other hand, it has been reported that the transesterification efficiency is dependent on the ratio of hydroxyl groups vs. ester groups within a crosslinked network. The BPAGMA contains a 1:1 ratio of hydroxyl groups and ester groups, enabling its good recyclability^48^. Due to insufficient understanding of the BPAGMA’s multifunctionality, some resin formulations adopted BPAGA only as a minor component, some formulations resulted in a less crosslinked network composed of BPAGMA, and some formulations possessed low ratio of hydroxyl groups vs. ester groups. In order to realize facile 3D printing using the BPAGMA-based resin while maintaining all the desired properties of UV-cured BPAGMA, 1,4-butanediol dimethacrylate (BDDMA) was mixed with the BPAGMA. On one hand, the BDDMA plays a role of a diluent, leading to better processability of the resin for additive manufacturing. On the other hand, BDDMA can be chemically crosslinked with BPAGMA upon UV light to retain a relatively uniform, highly crosslinked, and sterically hindered network similar to the network of UV-cured bulk BPAGMA. Along with an efficient photo-initiating system, the new resin can be readily cured upon shining UV light with controlled thickness as a function of UV exposure time (Fig. S1).

In our previous study^38^, the focus was on shape memory and recyclability of the BPAGMA resin. While the resin is UV curable, it is not 3D printable because the curing time, as long as 80 s, is not suitable for printing using the DLP system. The new resin is, therefore, called 3D printable and recyclable shape memory polymer, or 3D-RSMP for short. Conventionally, the UV-sensitive resin was cured within a mold which leads to limitations about inhomogeneous curing due to the oxygen inhibition on the surface and the shallow penetration depth of UV light. Additive manufacturing allows a layer-by-layer fabrication, hence, a more homogeneous network than that can be expected for UV-cured BPAGMA bulk with the highly photo-sensitive 3D-RSMP resin. Furthermore, we 3D printed lightweight architectures with varying geometries and systematically studied the multifunctionality of the lightweight architectures in the current study.

**Figure 2:**
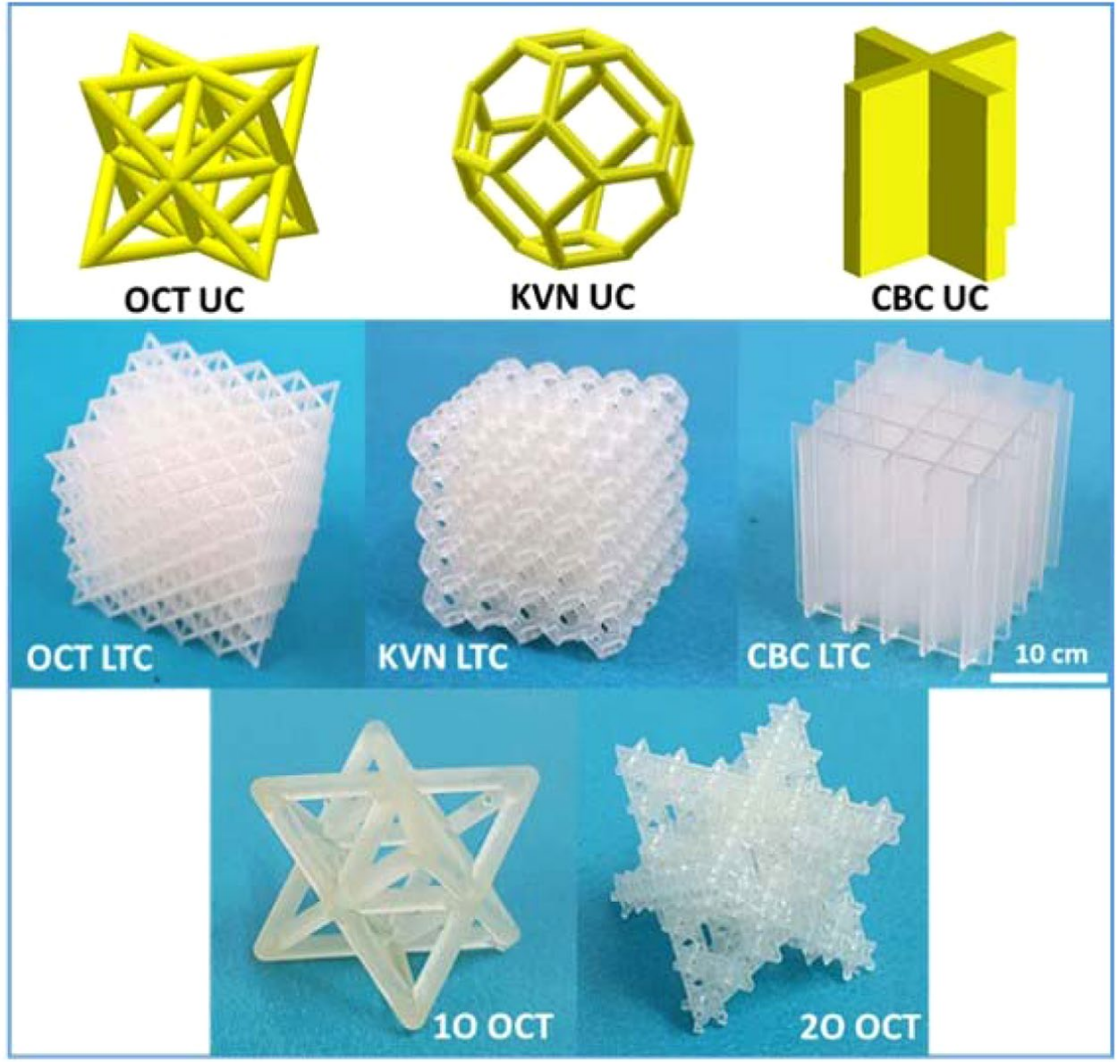
Three unit cell geometries have been drawn in Solidworks and then assembled to the corresponding microlattice structures. (Row 1: unit cells, from left to right: Octet unit cell (OCT UC); Kelvin unit cell (KVN UC); Cubic unit cell (CBC UC), Row 2: 3D printed microlattice structures, from left to right: Octet microlattice structure (OCT LTC), Kelvin microlattice structure (KVN LTC), and cubic microlattice structure (CBC LTC); Row 3: multi-length scale microlattices, from left to right: 1st order octet truss (1O OCT) and 2nd order octet truss (2O OCT)). (The scale bar applies to all the five lattice structures).

Two series of lightweight microlattice structures were printed. In the 1^st^ series of microlattices, an octet unit cell was selected as a representative of the stretching-dominated geometry, a Kelvin unit cell was selected as a bending-dominated geometry, and a cubic unit cell was selected due to its optimal unit cell orientation aligned with the predominant stress trajectories (Fig. 2). Each unit cell was then assembled into corresponding cubic microlattice (number of unit cells in each axis is 5 × 5 × 5) with varying densities. The 2^nd^ series was designed in order to mimic traditional lightweight materials (e.g. foams) which contain multi-length scale pores. Hence, we printed the first order octet and second order octet microlattices, and compared their compressive strength and modulus to investigate the effect of length scales. As for the efficiency of 3D printing with the 3D-RSMP resin, two cubic microlattice structures (30 × 30 × 30 mm) took about 70 min to print out, and only 26 min was required to print out 8 dogbone specimens at one time. Besides, several complex lattice structures with high resolution were 3D printed with the 3D-RSMP resin (Fig. S2a). To fully cure the 3D-RSMP resin, the 3D printed objects need to be post-cured for 1 h in a post-curing UV chamber (7.7 mW/cm^2^), leading to the color change of the 3D printed objects from light yellow to light orange (Fig. S2b,c). Depending on the geometry and dimensions of the printed object, the color change is less pronounced for thin-walled structures. The complete UV-curing is indicated by the FTIR analysis with respect to the disappearance of the double bond peak at 1635 cm^−1^ (Fig. S3). Zooming into the microlattice structures under an optical microscope, it is found that all the lateral members have smooth surfaces (Fig. S4a,c,e), and all the inclined or vertical members have layered surfaces (Fig. S4b,d,f) due to the layer-by-lay printing process. It is noted that for all the printed struts, there is no waviness nor varying cross-section. With vertical printing only, the thickness of each layer in the vertical wall of the cubic microlattice was measured as 0.15 mm, which was consistent with the printing parameter, suggesting high-quality and high-resolution DLP 3D printing with the 3D-RSMP resin.

### Characterization of the 3D-RSMP

A rectangular specimen made of the 3D-RSMP resin was printed and dynamic mechanical analysis (DMA) of the specimen shows that the 3D-RSMP has a glass transition temperature (*T*_*g*_) at 95 °C based on the peak of the tanδ (Fig. S5). The high stiffness of the 3D-RSMP is also suggested by the high storage modulus (*E’* = 2800 MPa) of the material at room temperature. More importantly, the *E’* of the 3D-RSMP remains relatively high (254 MPa) even at 150 °C, which is 55 °C above the *T*_*g*_. Compared to most other shape memory polymers without adequate recovery stress, the high storage modulus above *T*_*g*_ is an essential requirement for high recovery stress.

**Figure 3:**
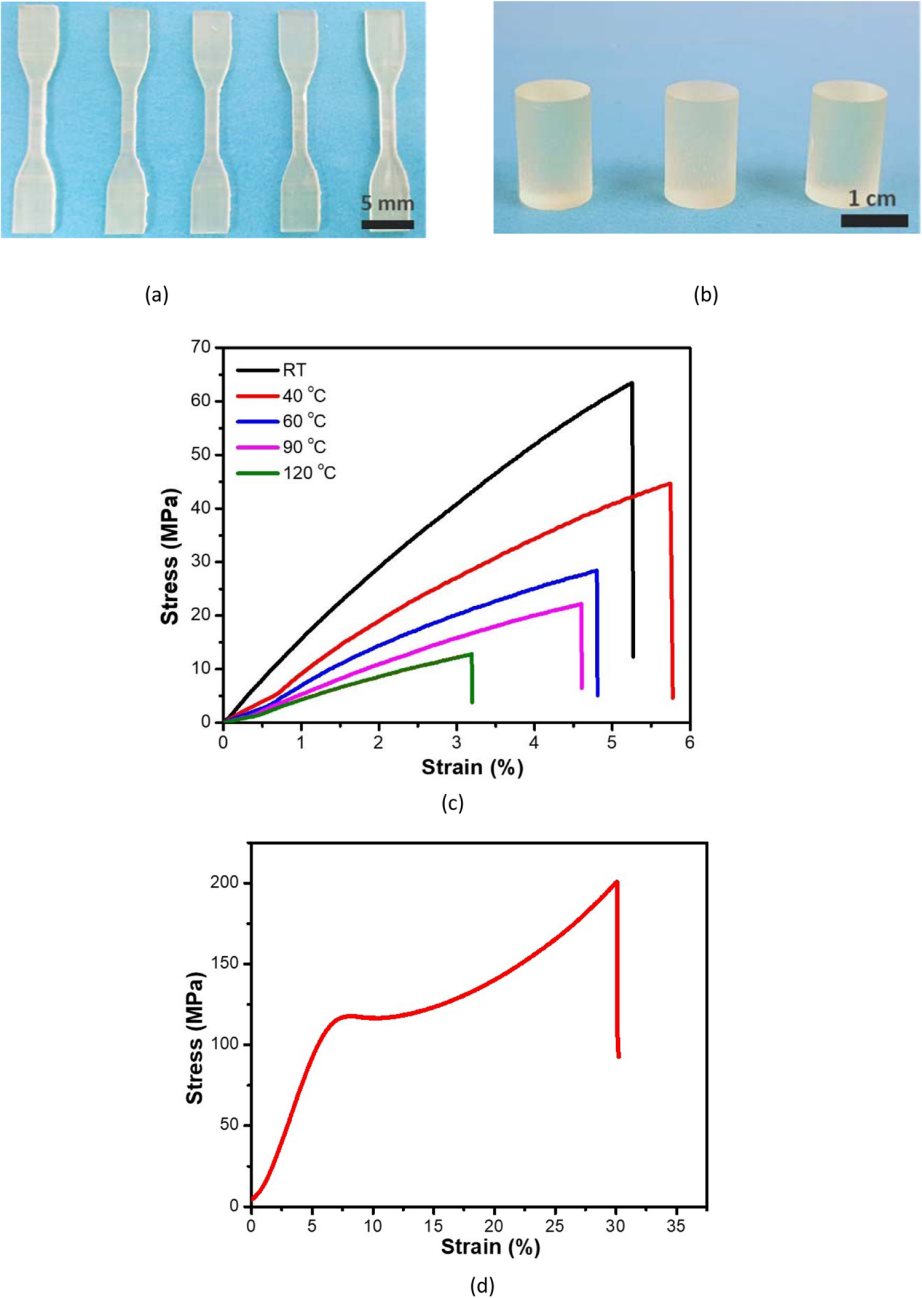
Mechanical properties of the 3D printed specimens. (**a**) 3D printed dogbone specimens for tensile tests. (**b**) 3D printed cylinders for compression tests. (**c**) Representative tensile stress vs. strain curves of the 3D printed dogbone specimens obtained from tensile test at various temperatures with a loading rate of 0.5 mm/min. (**d**) Representative room temperature compressive stress vs. strain curve of the 3D printed cylindrical specimens obtained from compression test at a loading rate of 1 mm/min.

The mechanical and thermal properties of the printed 3D-RSMP were then assessed. A series of dogbone specimens (neck length = 12.96 mm, width = 1.63 mm, thickness = 2.60 mm) were 3D printed and post-cured in a UV chamber (7.7 mW/cm^2^) for 1 h (Fig. 3a). Their tensile strength was measured by the MTS machine at various temperatures (room temperature (RT) to 120 °C). The representative stress vs. strain curve of the dogbone tensile tests indicate that the 3D-RSMP undergoes elastic deformation before fracturing at all the tested temperatures (Fig. 3c). The 3D-RSMP has a very high room temperature tensile strength (62 MPa) on par with traditional high-performance epoxy, and a high elastic modulus (1.46 GPa), suggesting its high stiffness (Table 1). The small ultimate tensile strain (5%) indicates that the 3D-RSMP is a brittle material (Table 1), similar to other load-bearing structural thermosets. As temperature rises, the tensile strength and modulus (slope of the tensile stress-tensile strain curve) decrease, and so is the ultimate tensile strain (Fig. 3c), except for the specimen at 40 °C. This “inconsistence trend” in ultimate tensile strain was also observed in other photopolymers^49^.

**Table 1:** Summary of mechanical strength of 3D printed dogbones and cylindrical specimens at room temperature.

Mechanical Properties of 3D printed objects	Test Results
Tensile Strength (MPa)^α^	62.0 ± 2.8
Ultimate Tensile Strain (%)^α^	5.0 ± 0.4
Compressive Strength (MPa)^β^	190.0 ± 7.7
Ultimate Compressive Strain (%)^β^	30.0 ± 0.7
Young’s Modulus (GPa)^α^	1.46 ± 0.07

^α^The results came from the uniaxial tension test of 3D printed dogbones.

^β^The results came from the uniaxial compression test of 3D printed cylinders.

The compressive behavior of the 3D-RSMP was obtained using 3D printed cylindrical specimens (Fig. 3b). At room temperature (glassy state), the cylindrical specimen goes through an elastic deformation first, then yields at about 8% strain, followed by strain softening and plastic flow, and eventually breaks at the end of the strain hardening region (Fig. 3d). This compression behavior is common for chemically cross-linked thermosets in the glassy state. The mechanical tests suggest that the 3D-RSMP has excellent mechanical properties and it is an appropriate material for fabricating high-performance microlattice structures.

### Mechanical properties of the 3D printed microlattices

**Figure 4:**
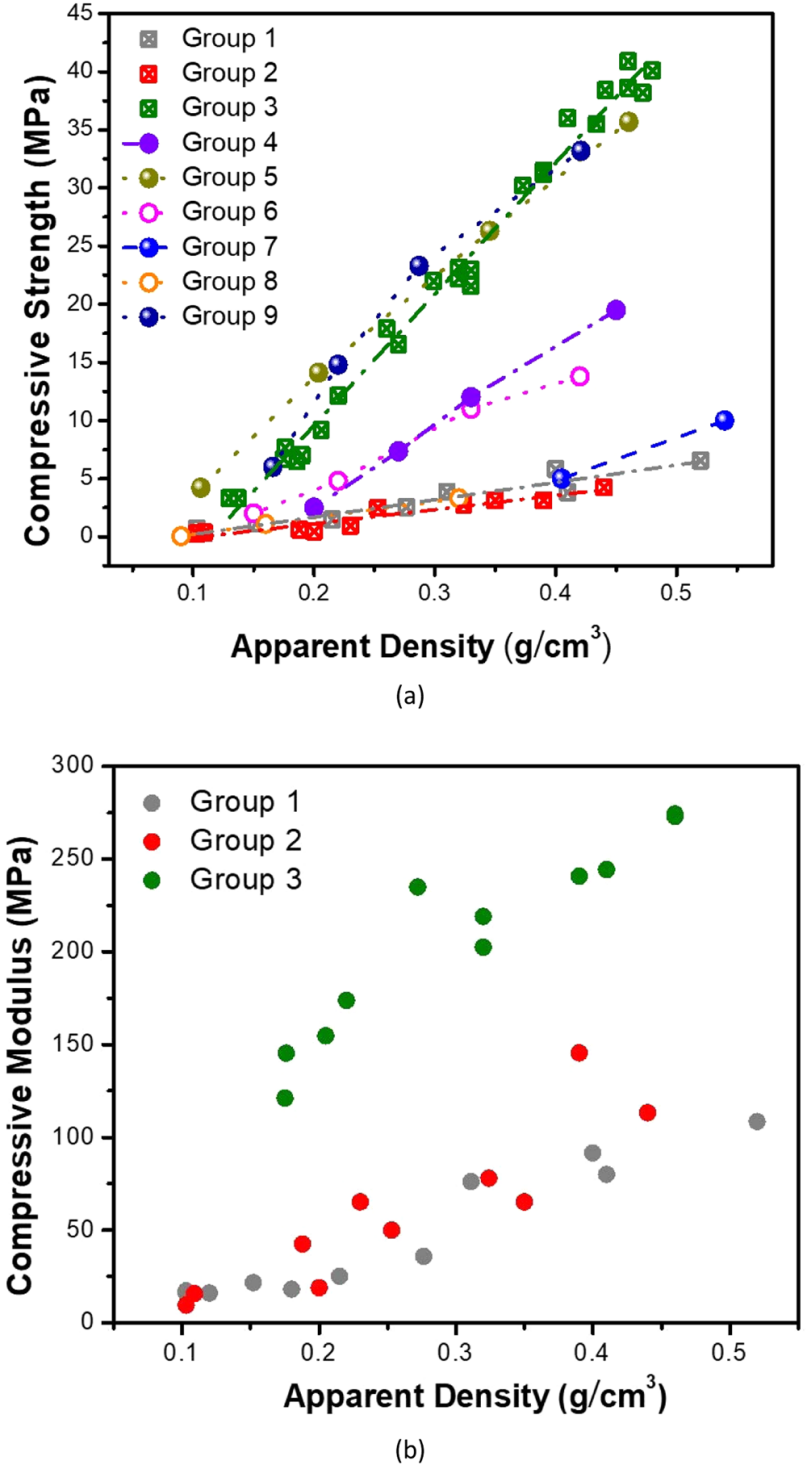
Mechanical properties of various microlattices upon compression. (**a**) Compressive strength vs. apparent density plots of various microlattices and foams. (**b**) Compressive modulus vs. apparent density plots of the three microlattices in this study.

To facilitate discussion, the various microlattices and foam materials are named as groups, here 11 groups as summarized in Table 2. With the apparent densities about 0.4 g/cm^3^ (weight of the microlattice divided by the overall volume of the microlattice, including the volume of the voids), all the three microlattices (Groups 1–3) went through compression to failure (Fig. S6a). Both the octet microlattice and Kelvin microlattice collapsed with very similar loading (Fig. S6b). The cubic microlattice exhibits a much higher strain-energy storage capability than the other two microlattices due to the optimal geometric orientation (each unit cell can also be treated as a square cylinder under uniaxial compression), and the cubic microlattice collapsed due to brittle fracture. Further assessment was made to the dependence of mechanical strength on the microlattice apparent density. All the statistical results are summarized and plotted, and representative values are also listed (Table S1). The compressive strength of all the three microlattices increases linearly with the density. The octet microlattice shows a slightly better compressive strength than the Kelvin microlattice throughout the density range from 0.1 to 0.45 g/cm^3^, which is consistent with the theoretical prediction (Figs. 4a and S7a). The compressive strength of the octet microlattice (Group 1) within the tested density range is comparable to commercial polyurethane foams (Group 8) (Figs. 4a and S7a)^50^.

**Table 2:** Summary of the lightweight architectures and the corresponding group number.

Group #	Lightweight Architecture
1	Octet Microlattice (this study)
2	Kelvin Microlattice (this study)
3	Cubic Microlattice (this study)
4	Commercial Cubic Microlattice
5	Titanium Octet Truss^α^
6	Rigid Polyurethane Foam^β^
7	Aluminum Foam^γ^
8	Commercial Polyurethane Foam^δ^
9	Ceramic Composite Microlattice A
10	First Order Octet Microlattice (this study)
11	Second Order Octet Microlattice (this study)

^α^The apparent density of the Ti octet lattice was calculated by assuming that the density of the bulk metal alloy (Ti-6Al-4V) is 4.43 g/cm^3^.

^β^The compressive strength of the rigid polyurethane foam is estimated based on a reported plot.

^γ^The compressive strength of the aluminum foam is estimated based on a reported plot of compressive strength vs. relative density, and the density of the foam is estimated by assuming the bulk density of the metal alloy is 2.7 g/cm^3^.

^δ^The compressive strength of the commercial polyurethane foam is the reported mean yield strength.

Throughout the density range, the cubic microlattice (Group 3) exhibits much higher compressive strength than octet (Group 1) and Kelvin (Group 2) microlattices. The geometric effect is clearly reflected by the enhanced mechanical strength of the Group 3 as compared to a traditional rigid polyurethane foam (Group 6), which is composed of a high-performance polymer but lacks the structural efficiency (Fig. 4a)^51^. The same cubic microlattice structure made of a commercial 3D printing resin (Group 4), which does not have high mechanical strength (Veriguide, tensile strength 28.5 MPa, elastic modulus 1.14 GPa), was also tested. It is found that the mechanical strength of the commercial cubic microlattice (Group 4) is comparable to that of the rigid polyurethane foam (Group 6) but lower than the cubic microlattice (Group 3) made of the 3D-RSMP (Fig. 4a), confirming that optimal mechanical strength of the microlattice is governed by both the property of the 3D printable resin and the geometry.

Octet truss made of titanium alloy (Group 5) and ceramic composite microlattice A (Group 9, polymer lattice with Al_2_O_3_ coating) have shown excellent mechanical strength^23,24^. The compressive strength of the cubic microlattice (Group 3) is slightly smaller than the titanium octet truss within the density range from 0.1 to 0.3 g/cm^3^ (Figs. 4a and S7a), and yet when the density ranges from 0.3 to 0.5 g/cm^3^, the cubic microlattice shows comparable compressive strength to the titanium octet truss and ceramic composite microlattice A structures. As compared to other metallic microlattices, the cubic microlattice (Group 3) exhibits even better mechanical strength^24,25^. In comparison, the traditional aluminum foam (Group 7), which is widely used in lightweight structures, cannot compete with the structural efficient cubic microlattice (Group 3)^52^.

The dependence of the compressive modulus on the apparent microlattice density shows that the octet (Group 1) and Kelvin (Group 2) microlattices have comparable compressive moduli within the tested density range. Meanwhile, the cubic microlattice (Group 3) shows much higher compressive modulus than the other two do (Fig. 4b). The relative modulus of the microlattices (compressive modulus of the microlattices against the elastic modulus of the 3D-RSMP) as a function of relative density (apparent density of the microlattices against the bulk density of the 3D-RSMP) was also plotted (Fig. S7b), leading to the same conclusion.

The dependence of the mechanical properties on the apparent density for both 1^st^ order octet truss (Group 10) and 2^nd^ order octet truss (Group 11) was also studied. Theoretically, the 2^nd^ order microlattice should possess higher compressive strength than the 1^st^ order microlattice if both of them undergo buckling failure mechanism. However, the experimental results show that there is no significant difference between them regarding the compressive strength within the tested density range (0.1 to 0.3 g/cm^3^) (Fig. S8a), although the Group 10 has a slightly higher modulus than the Group 11 does (Fig. S8b). The deviation from the theoretical model may be due to the brittle fracture failure mode of the two types of microlattices. It is seen that the fracture occurs mainly at the nodes rather than on the struts, resulting in brittle fracture instead of buckling (Fig. S8c). The reason that structural failure is dominated by fracture instead of buckling may be due to the fact that the octet microlattice is a stretch-dominated structure, i.e., most members or ribs are subjected to tensile stress. However, as given in Table 1, the 3D-RSMP is very brittle under tension, leading to fracture in the nodal areas, instead of rib buckling.

### Shape memory effect of the 3D-RSMP and the cubic microlattices (Group 3)

**Figure 5:**
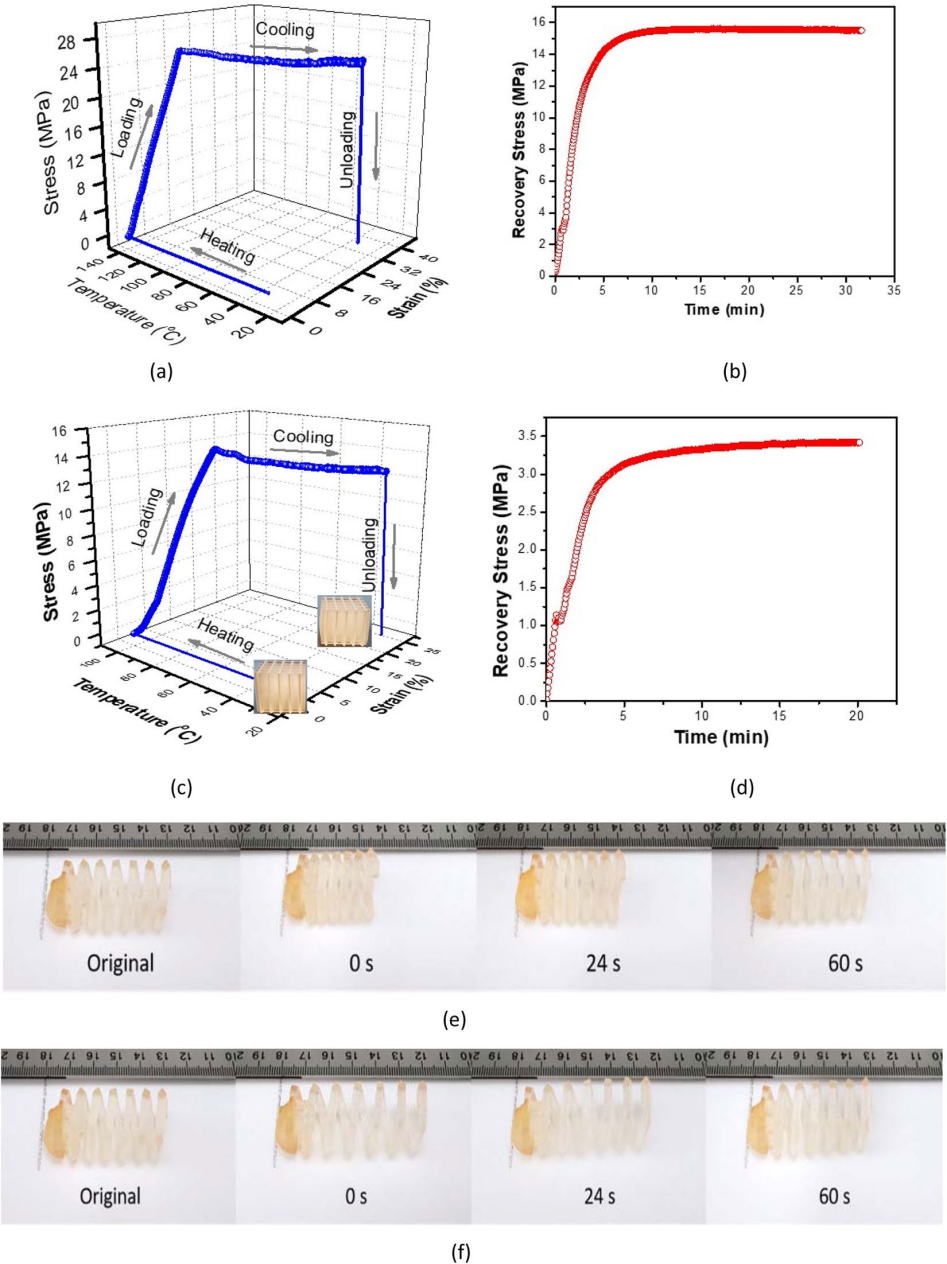
Shape memory behavior and recovery stress of the 3D-RSMP and the cubic microlattice. (**a**) 4-step hot programming of the 3D printed cylinder: (1) heating to 150 °C, (2) loading to 17% strain, (3) cooling under constant stress, and (4) unloading to zero after cooling. (**b**) Recovery stress as a function of time based on a fully constrained 3D printed cylindrical specimen (14% pre-strain) at 150 °C. (**c**) 4-step hot programming of the 3D printed cubic microlattice at 100 °C. (**d**) Recovery stress vs. time based on a fully constrained cubic microlattice (8.8% pre-strain) at 100 °C. (**e**) Free shape recovery of a compression programmed 3D-printed spring upon heating. (**f**) Free shape recovery of a stretch programmed 3D-printed spring upon heating.

Although high-performance metallic microlattices have been fabricated, their shape memory effect has not been developed, limiting further advanced structural applications. This issue can be resolved by applying the 3D-RSMP to print microlattices, and the shape memory effect of the 3D-RSMP was first evaluated. Besides the good mechanical strength, a good shape memory polymer for structural application is determined by three important factors, the shape fixity ratio (***F***), which implies how much strain can be memorized; free shape recovery ratio (***R***), which quantifies how much strain can be recovered; and the recovery stress, which suggests how much force can be exerted on to an object during the recovery process. In order to measure ***F***, ***R*** and the recovery stress, printed 3D-RSMP specimens (cylinders or microlattices) was first programmed by following a 4-step procedure: (1) heating up the system, (2) loading at rubbery temperature, (3) cooling to glassy state while holding the stress constant, and (4) unloading (Fig. 5a). To briefly introduce this process, a 3D printed cylinder (diameter 8.95 mm and height 13.92 mm) made of the 3D-RSMP resin was compressed by the MTS machine at 150 °C in an oven which was pre-heated for 1 h, and a subsequent cooling step was conducted to freeze the motion of the polymer chain segments and fix the temporary shape. It is shown that about 24 MPa was needed to compress the cylinder at 150 °C for 17% strain. The stress was maintained the same at zero loading rate during the cooling process and became zero after removing the external load at room temperature (the 4th step – unloading) (Fig. 5a). ***F*** was obtained by dividing the height of the cylinder after unloading by the height of the cooled cylinder under load (Eq. 2). The shape recovery ratio ***R*** was measured by performing a free shape recovery test at 150 °C using the programmed cylinder (Eq. 3). The cylinder exhibits excellent shape memory properties, suggested by almost 100% ***F*** and 97% ***R***.

Recovery stress was obtained by heating a programmed cylinder at 150 °C whose two ends were confined by the MTS fixtures. The MTS system was pre-heated at 150 °C for 1 h to avoid any interference from the thermal expansion due to heating the steel fixtures. It shows that the programmed but confined cylinder has a quick recovery stress response once it was heated up at 150 °C, and the recovery stress reached its plateau after approximately 8 min and stayed at its maximum for more than 30 min without any loss of the stress (Fig. 5b). Upon multiple measurements, the maximum recovery stress of the cylinder is 12.1 ± 2.5 MPa. Based on previous literatures, there are only a few thermoset shape memory polymers with recovery stress above 10 MPa in the rubbery state and in bulk form.

The shape memory test was then conducted with the cubic microlattice, which has excellent mechanical strength and a great potential for structural utilization. Due to the reduced amount of materials and voids in the structure, the cubic microlattice is more likely to fracture at a higher temperature before an adequate deformation can be made during the hot compression programming. Hence, the same 4-step hot programming was performed with a cubic microlattice (0.46 g/cm^3^) at 100 °C, which is right above the *T*_*g*_ but imparts the microlattice with higher modulus and larger ultimate compressive strain as compared to the high-temperature programming at 150 °C. After the 4-step hot programming, the microlattice became a shorter and wider microlattice compared to the initial structure (Fig. 5c). The free shape recovery test suggests that the microlattice can reach 83% strain recovery at 100 °C. To achieve a higher ***R***, the specimen can be either held longer time at 100 °C, or heated up to 150 °C with shorter holding time^53^. In this study, the temperature was raised to 150 °C, which was completely above the *T*_*g*_ region, leading to 95% ***R***. Recovery stress of the programmed microlattice was measured by fully confining it using the MTS fixtures at 100 °C. A quick stress response similar to the solid cylinder was seen with the microlattice (8.8% pre-strain), which reaches around 3 MPa at about 5 min. Due to the lower density than the solid cylinder, the microlattice takes longer to stabilize at the plateau of 3.4 MPa (Fig. 5d), which is larger than the maximum recovery stress of many solid shape memory polymers. Likewise, the cubic microlattice is the first shape memory lattice structure with a decent recovery stress and high mechanical strength comparable to metallic microlattices.

Due to the high stiffness of the 3D-RSMP, most of the 3D/4D printed structures need a large stress to program with a relatively small strain (less than 15%), making it difficult to visually observe the shape memory effect. However, the shape memory effect of the 3D-RSMP can still be clearly demonstrated with 3D/4D printed flexible structures, like springs. A spring was printed using the 3D-RSMP resin, and it was programmed under both compression and tension. Under the compression programming, the spring was able to fix about 20% strain (Fig. 5e). It exhibited the shape recovery effect upon heating and was able to recover the full strain after 1 min. A reversed shape memory behavior of the spring could also be achieved by performing a stretch programming. It was similar to the compression programming. The spring could fix about 20% strain after the stretch programming, and it recovered all the 20% strain upon heating for about 1 min (Fig. 5f). A thermal contraction behavior of the spring could be seen as a result of stretch programming.

### Recycling of the 3D printed microlattices

The application of microlattices is for the purpose of developing cost and energy-efficient aircraft, cars, ships, etc. As a result, at the end of the life cycle, the microlattices should be recycled instead of burned or landfilled, which is typical for traditional thermoset-based lightweight materials.

**Figure 6:**
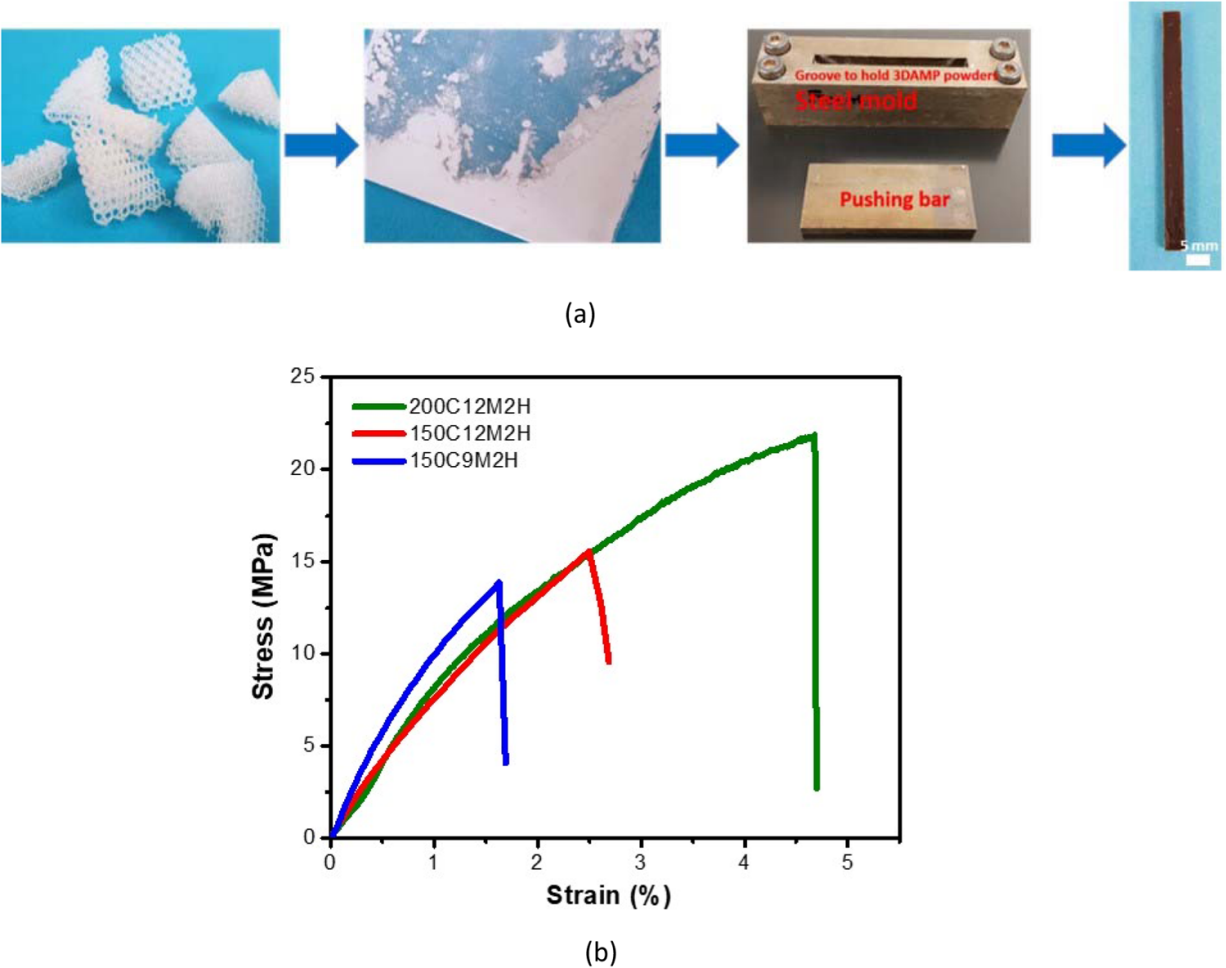
Recycling of the crushed microlattices. (**a**) A recycling process is described: broken and failed shape memory microlattices were crushed into powders via ball milling; a steel mold was used for recycling milled powders of 3D printed microlattice structures under varying conditions. A mechanical test was performed on the remolded rectangular specimen made of the milled powders. (**b**) Typical tensile stress vs. strain curves of the remolded rectangular specimens obtained under varying conditions ((200C12M2H represents molding at 200 °C and under 12 MPa pressure for 2 h; 150C12M2H represents molding at 150 °C and under 12 MPa pressure for 2 h; and 150C9M2H represents molding at 150 °C and under 9 MPa pressure for 2 h) with a loading rate = 0.5 mm/min at room temperature.

Therefore, the recyclability of the 3D-RSMP and the microlattices was investigated. Briefly, the crushed microlattice structures (Fig. 6a) were first broken into powders via ball milling (10 g for 20 h), and then loaded in a steel mold (Fig. 6a) and remolded under high temperature and high pressure for 2 h. The effect of recycling conditions on the recycling efficiency of the UV-cured BPAGMA has been systematically studied, showing that the recycling efficiency in terms of the interfacial transesterification reaction is proportional to the recycling temperature and pressure^38^. High pressure above 6 MPa is usually applied to allow adequate surface contact between milled particles, and the temperature needs to be above the T_g_ of the thermoset for the diffusion of chain segments across the contact interfaces. Hence, we performed the solid-state recycling under three recycling conditions, and the recycling efficiency is characterized by the tensile strength of the recycled specimen against the original specimens. Upon the uniaxial tension test, the specimens recycled from the three conditions undergo elastic deformation (Fig. 6b). It was found that when the compressive pressure was increased from 9 MPa to 12 MPa, the tensile strength of the recycled specimens increased slightly from 12.7 to 13.5 MPa (Table 3), indicating that the compressive pressure has limited effect on the recycling results. In contrast, the recycling temperature is crucial to obtain good recycling results. By increasing the recycling temperature from 150 °C to 200 °C, the tensile strength of the recycled specimen increased from 13.5 MPa to 17.6 MPa (Table 3). Higher recycling temperature results in higher reactivity of transesterification reactions and higher chain mobility, and thus, the recycling efficiency can be improved. As compared to the UV-cured BPAGMA (25.5 MPa), the tensile strength of the recycled 3D-RSMP is decreased, mainly due to the introduction of the BDDMA to the resin, which increased the ester bond/hydroxyl group ratio to above 1^38^. According to previous studies, the highest transesterification efficiency can be reached when the ester/hydroxyl group ratio is 1^48^. Meanwhile, the elastic modulus (0.98 ± 0.14 GPa) of the recycled 3D-RSMP is still relatively high. One of the future directions will be focusing on systematic studies of the catalytic effects on improving the recycling efficiency of the printable and recyclable 3D-RSMP.

**Table 3:** Tensile test results of recycled 3D-RSMP under varying recycling conditions.

Recycling Condition	Tensile Strength (MPa)	Ultimate Tensile Strain (%)	Young’s Modulus (GPa)
200C12M2H^α^	17.6 ± 3.1	3.0 ± 1.3	0.98 ± 0.14
150C12M2H	13.5 ± 2.0	3.5 ± 1.0	0.68 ± 0.04
150C9M2H	12.7 ± 1.1	1.8 ± 0.2	0.80 ± 0.15

^α^C represents °C; M represents MPa; H represents hour.

## Discussion

A unique 3D-RSMP resin for universal DLP 3D printing has been developed. The 3D-RSMP has mechanical properties (tensile strength = 62.0 MPa and elastic modulus = 1.46 GPa at room temperature) comparable to those of the best commercial DLP resins without multifunctionality. The mechanical strength of the 3D-RSMP is also higher than most current 3D printable shape memory polymers and self-healable 3D printing polymers. The shape memory effect and especially the shape recovery stress of the 3D-RSMP is much higher than current 3D printable shape memory polymers. To our knowledge, the 3D-RSMP is the only 3D printable and recyclable shape memory polymer with recovery stress larger than 10 MPa (Table S2)^33–35,46,47,49,54^. The development of the 3D-RSMP enables fabrication of multifunctional lightweight architectures (MLAs).

By applying the 3D-RSMP to printing microlattices, the effects of geometry and length scales on the mechanical properties and shape memory effect were studied, and it showed that the cubic microlattice has the highest mechanical strength with comparable or even higher specific compressive strength than metallic microlattices and ceramic microlattices without shape memory effect. In addition, the cubic microlattices exhibit good shape memory properties and are able to produce a decent recovery stress (3.4 MPa), which is larger than many bulk shape memory polymers. Owing to the recyclability of the cubic microlattice, sustainable lightweight structures can be achieved through its whole life cycle.

Future work will be focusing on improving the recycling efficiency of the 3D-RSMP and the microlattice, and further optimizing the geometry through topological optimization or biomimicry in order to obtain microlattices with higher mechanical strength and shape memory effect for advanced structural and engineering applications.

## Materials and Methods

### Experimental design

The objective of this study is to develop a 3D printable thermoset polymer integrated with high strength, high stiffness, high shape recovery ratio, high recovery stress, and good recyclability. To this purpose, a systematic experimental program was designed. We started by selecting the resin which has high mechanical strength and stiffness once cured, and also added multifunctionality such as shape memory, recyclability, and 3D printability. We then characterized the printed specimens with its mechanical and functional properties. Once the properties came to the design level, we started printing microlattices with various unit cells and length scales, and tested their mechanical and shape/stress memory effect. We finally examined the recyclability of crushed microlattices. For all the tests, at least three specimens were used and the average and standard deviation were reported in the Tables; for line graphs, only representative test results were plotted.

### Resin formulation and 3D/4D printing

In this study the 3D printable and recyclable shape memory polymer (3D-RSMP) resin was prepared by mixing 2-hydroxy-2-methylpropiophenone (3.5 wt%), bis(2,4,6-trimethylbenzoyl)-phenylphosphineoxide (1 wt%), triethylamine (0.5 wt%), 1,4-butanediol dimethacrylate (20 wt%) in bisphenol A glycerolate dimethacrylate (BPAGMA, 200 g) at 72 °C for 4 hours. All the reagents were purchased from Sigma Aldrich and used as received.

All the structures were designed using Solidworks 2016, processed by Composer 1.2.5, and printed by the Asiga Pico 2 39 SLA 3D printer (28 mW/cm^2^, 385 nm) using the as-prepared 3D-RSMP resin with 0.15 mm layer thickness.

### Compositional analysis, thermal analysis, and thermomechanical analysis

The compositions of the resin and the 3D-RSMP were analyzed using FTIR (Bruker Alpha FTIR Spectrometer) with the scanning range from 400 to 4000 cm^−1^. The IR spectra were obtained by measuring the resin and the ball milled 3D-RSMP powder in the same sample holder and fixed by a press to maintain the same sample dimensions.

Thermomechanical test of 3D printed 3D-RSMP thin sheet specimens (18.6 × 6.8 × 1.4 mm) was conducted using model Q800 DMA (TA Instruments, DE, USA) in the multi-frequency-strain mode with 3 °C/min temperature ramp rate, 0 to 180 °C temperature scan range, 10 µm amplitude, and 1 HZ frequency.

### Tensile strength and compressive strength tests

3D printed dogbone specimens (neck length = 12.96 mm, width = 1.63 mm, and thickness = 2.60 mm) were used for the tensile strength test at various temperatures. All the tensile strength tests were performed by the mechanical test system (MTS) machine (Alliance RT/5, MTS, USA) with 0.5 mm/min loading rate.

3D printed cylindrical specimens (diameter = 8.95 mm and height = 13.92 mm) and various cube microlattice structures were used for the compression test. All the compression tests were performed by the mechanical test system (MTS) machine (QTEST 150 machine, MTS, USA) with 1 mm/min loading rate. All the mechanical tests were repeated for three times to obtain the average value and standard deviation.

### Shape memory effect and recovery stress tests

The shape memory effect of the 3D printed cylinders (diameter = 8.95 mm and height = 13.92 mm) and cubic microlattices (13.88 mm × 13.89 mm × 14.05 mm) were performed at a temperature above the *T*_*g*_ (150 °C for the cylinder and 100 °C for the microlattices) using the MTS machine with a loading rate of 1 mm/min, followed by cooling down the whole MTS system to room temperature while holding the stress constant. After unloading at room temperature, the compression programming process was completed. Free shape recovery test was conducted by heating both the programmed cylinder and cubic microlattice to 150 °C without any external constraint. Shape fixity ratio (***F***) and shape recovery ratio (***R***) were calculated using Eqs 2 and 3, respectively:

2 $${\boldsymbol{F}}=\frac{{\varepsilon }_{f}}{{\varepsilon }_{l}}\times 100 \% $$

3 $${\boldsymbol{R}}=\frac{{{\boldsymbol{\varepsilon }}}_{{\boldsymbol{f}}}-{{\boldsymbol{\varepsilon }}}_{{\boldsymbol{r}}}}{{{\boldsymbol{\varepsilon }}}_{{\boldsymbol{f}}}}\times 100{\rm{ \% }}$$

where *ɛ*_*l*_ is the measured strain before load removal, *ɛ*_*f*_ is the fixed strain after load removal, and *ɛ*_*r*_ is the residual strain after recovery.

A 3D/4D printed spring was used for the demonstration of free shape recovery. The spring was first heated with a heat gun, and then compressed or stretched as compression or tension programming at high temperature. The deformed spring was then immersed in a cool water bath to fix the temporary shape. Free shape recovery was then observed by re-heating the deformed spring for 1 min using the heat gun (Video S1).

To measure the recovery stress, the programmed cylinder or cubic microlattice were fully constrained by the fixtures of the MTS machine (QTEST 150 machine, MTS, USA) in a pre-heated oven (150 °C for the programmed cylinder and 100 °C for the programmed microlattices) for 1 hour so that the thermal expansion of the metal fixtures can be avoided. Once the specimen was confined, the data collection was started to record the stress as a function of time. All the tests of shape recovery ratio, fixity, and recovery stress were repeated at least for three times to obtain the average value and standard deviation.

### Recycling

The crushed and failed 3D printed cube microlattices (10 g) were first manually broken into small pieces and then added into the ceramic jars of the ball milling machine (Across International PQ-N2 Planetary, Livingston, New Jersey, USA). The broken pieces were then ground into particles through ball milling at room temperature for 20 h. The particles were added into a steel mold (Fig. 6a), and the recycling process was carried out by applying a pressure (12 MPa or 9 MPa) on the steel mold at a temperature 200 °C or 150 °C for 2 h. By removing the bottom of the steel mold and compressing the pushing bar, a dark brown specimen (4.21 mm × 4.98 mm × 59.99 mm) was obtained. The recycling efficiency was assessed using Eq. 4:

4 $$Recycling\,Efficiency=\frac{{\sigma }_{R}}{\,{\sigma }_{o}}\times 100 \% $$

where *σ*_*R*_ is the tensile strength of the recycled 3D-RSMP and *σ*_0_ is the tensile strength of the initial 3D-RSMP before recycling. All the mechanical tests were repeated for three times to obtain the average value and standard deviation.

### Optical microscopic analysis

Preliminary visualization of the printed microlattices was conducted using the AmScope Binocular Stereo Microscope and the images were collected and analyzed using AmScope 3.7, which was pre-calibrated.

## Supplementary information

Supplementary Information

Video S1

## Data Availability

All other data are available from the authors upon reasonable request.

## References

[1] Ishai O, Hiel C (1992). Damage tolerance of a composite sandwich with interleaved foam core. Journal of Composite Technology and Research.

[2] Reddy TY, Wen HM, Reid SR, Soden PD (1998). Penetration and perforation of composite sandwich panels by hemispherical and conical projectiles. ASME Journal of Pressure Vessel Technology.

[3] Mines RAW, Worrall CM, Gibson AG (1998). Low velocity perforation behaviour of polymer composite sandwich panels. International Journal of Impact Engineering.

[4] Vaidya UK, Nelson S, Sinn B, Mathew B (2001). Processing and high strain rate impact response of multi-functional sandwich composites. Composite Structures.

[5] Aktay L, Johnson AF, Holzapfel M (2005). Prediction of impact damage on sandwich composite panels. Computational Materials Science.

[6] Hou WH, Zhu F, Lu GX, Fang DN (2010). Ballistic impact experiments of metallic sandwich panels with aluminum foam core. International Journal of Impact Engineering.

[7] Li G, Muthyala VD (2008). Impact characterization of sandwich structures with an integrated orthogrid stiffened syntactic foam core. Composites Science and Technology.

[8] Shutov, F. A. Syntactic polymer foams. In Klempner D. and Frisch K. C. ed. *Handbook of polymer foams and foam technology*, Hanser Publishers: 355–374 (1991).

[9] Griffith G (2002). Carbon foam: a next-generation structural material. Industrial Heating.

[10] Bardella L, Genna F (2001). On the elastic behavior of syntactic foams. International Journal of Solids and Structures.

[11] Evans AG, Hutchinson JW, Ashby MF (1998). Multifunctionality of cellular metal systems. Progress in Materials Science.

[12] Van Vuure, A. W. Composite panels based on woven sandwich-fabric preforms. Ph.D. Thesis, Katholieke Universiteit Leuven, Belgium (1997).

[13] Hosur MV, Abdullah M, Jeelani S (2005). Manufacturing and low-velocity impact characterization of foam filled 3-D integrated core sandwich composites with hybrid face sheets. Composite Structures.

[14] Hasebe RS, Sun CT (2000). Performance of sandwich structures with composite reinforced core. J. Sandw. Struct. Mater..

[15] Li G, Chakka VS (2010). Isogrid Stiffened Syntactic Foam Cored Sandwich Structure under Low Velocity Impact. Compos. Part. A Appl. Sci. Manuf..

[16] Gibson JL, Ashby FM (1982). The mechanics of three-dimensional cellular materials. Proc. Royal Soc. Lond. A.

[17] Gibson, J. L. & Ashby, F. M. Cellular Solids: Structure and Properties. Cambridge Univ. Press, Cambridge (2001).

[18] Gibson JL (2003). Cellular Solids. MRS Bulletin.

[19] Lan X (2009). Fiber reinforced shape memory polymer composite and its application in a deployable hinge. Smart Mater. Struct..

[20] Li G, Nettles D (2010). Thermomechanical Characterization of a Shape Memory Polymer Based Self-Repairing Syntactic Foam. Polymer.

[21] Lu L, Fan J, Li G (2016). Intrinsic Healable and Recyclable Thermoset Epoxy Based on Shape Memory Effect and Transesterification Reaction. Polymer.

[22] Shi Q, Yu K, Dunn ML, Wang T, Qi HJ (2016). Solvent assisted pressure-free surface welding and reprocessing of malleable epoxy polymers. Macromolecules.

[23] Bauer J, Hengsbach S, Tesari I, Schwaiger R, Kraft O (2014). High-strength cellular ceramic composites with 3D microarchitecture. Proc. Natl. Acad. Sci. USA.

[24] Dong L, Deshpande V, Wadley H (2015). Mechanical response of Ti-6Al-4V octet-truss lattice structures. Int. J. Solids. Struct..

[25] Li Q, Chen EY, Bice DR, Dunand DC (2008). Mechanical properties of cast Ti-6Al-4V lattice block structures. Metall. Mater. Trans. A.

[26] Torrents A, Schaedler T, Jacobsen A, Carter W, Valdevit L (2012). Characterization of nickel-based microlattice materials with structural hierarchy from the nanometer to the millimeter scale. Acta Mater..

[27] Tibbits S (2014). 4D printing: multi‐material shape change. Architectural Design.

[28] Lu H, Yu K, Liu Y, Leng J (2010). Sensing and actuating capabilities of a shape memory polymer composite integrated with hybrid filler. Smart. Mater. Struct..

[29] Ware T (2012). Three-Dimensional Flexible Electronics Enabled by Shape Memory Polymer Substrates for Responsive Neural Interfaces. Macromol. Mater. Eng..

[30] Lendlein A, Langer R (2002). Biodegradable, elastic shape-memory polymers for potential biomedical applications. Science.

[31] Liu Y, Du H, Liu L, Leng J (2014). Shape memory polymers and their composites in aerospace applications: a review. Smart Mater. Struct..

[32] Cianchetti M (2014). Soft robotics technologies to address shortcomings in today’s minimally invasive surgery: the STIFF-FLOP approach. Soft Robot..

[33] Wallin T (2017). Click chemistry stereolithography for soft robots that self-heal. J. Mater. Chem. B.

[34] Ge Q (2016). Multimaterial 4D printing with tailorable shape memory polymers. Sci. Rep..

[35] Zarek M (2016). 3D printing of shape memory polymers for flexible electronic devices. Adv. Mater..

[36] Anthamatten M, Roddecha S, Li J (2013). Energy storage capacity of shape-memory polymers. Macromolecules.

[37] Fan J, Li G (2018). High enthalpy storage thermoset network with giant stress and energy output in rubbery state. Nat. Commun..

[38] Li A, Fan J, Li G (2018). Recyclable thermoset shape memory polymers with high stress and energy output via facile UV-curing. J. Mater. Chem. A.

[39] Yakacki CM (2008). Strong, tailored, biocompatible shape‐memory polymer networks. Adv. Funct. Mater..

[40] Kloxin CJ, Scott TF, Adzima BJ, Bowman CN (2010). Covalent adaptable networks (CANs): a unique paradigm in cross-linked polymers. Macromolecules.

[41] Li A, Zhang D (2016). Synthesis and characterization of cleavable core-cross-linked micelles based on amphiphilic block copolypeptoids as smart drug carriers. Biomacromolecules.

[42] Ihsan AB (2016). Self-healing behaviors of tough polyampholyte hydrogels. Macromolecules.

[43] Lu L, Pan J, Li G (2017). Recyclable high-performance epoxy based on transesterification reaction. J. Mater. Chem. A.

[44] Zhang P, Li G (2016). Advances in healing-on-demand polymers and polymer composites. Prog. Polym. Sci..

[45] Postiglione G, Alberini M, Leigh S, Levi M, Turri S (2017). Effect of 3D-printed microvascular network design on the self-healing behavior of cross-linked polymers. ACS Appl. Mater. Interfaces.

[46] Shi Q (2017). Recyclable 3D printing of vitrimer epoxy. Mater. Horizons.

[47] Kuang X (2018). 3D Printing of Highly Stretchable, Shape-Memory, and Self-Healing Elastomer toward Novel 4D Printing. ACS Appl. Mater. Interfaces.

[48] Capelot M, Montarnal D, Tournilhac F, Leibler L (2012). Metal-catalyzed transesterification for healing and assembling of thermosets. Journal of the American Chemical Society.

[49] Yu R (2017). Three-dimensional printing of shape memory composites with epoxy-acrylate hybrid photopolymer. ACS Appl. Mater. Interfaces.

[50] Patel PS, Shepherd DE, Hukins DW (2008). Compressive properties of commercially available polyurethane foams as mechanical models for osteoporotic human cancellous bone. BMC Musculoskelet. Disord..

[51] Traeger R (1967). Physical properties of rigid polyurethane foams. J. Cell. Plast..

[52] Koza E, Leonowicz M, Wojciechowski S, Simancik F (2004). Compressive strength of aluminium foams. Mater. Lett..

[53] Li G, Xu W (2011). Thermomechanical behavior of thermoset shape memory polymer programmed by cold-compression: testing and constitutive modeling. J. Mech. Phys. Solids..

[54] Zhang B, Kowsari K, Serjouei A, Dunn ML, Ge Q (2018). Reprocessable thermosets for sustainable three-dimensional printing. Nat. Commun..

