# Supplementary material for: 4D Printing of Recyclable Lightweight Architectures Using High Recovery Stress Shape Memory Polymer
Source: Sci Rep. 2019 May 20;9:7621. doi: 10.1038/s41598-019-44110-9 (PMC6527608; doi:10.1038/s41598-019-44110-9)
Supplement: Supplementary file 1 — Supplementary Information [file 41598_2019_44110_MOESM1_ESM.docx]

Supporting Information

4D Printing of Recyclable Lightweight Architectures Using High Recovery Stress Shape Memory Polymer

*Ang Li, Adithya Challapalli, and Guoqiang Li**

Department of Mechanical & Industrial Engineering, Louisiana State University, Baton Rouge, Louisiana 70803, USA

E-mail: *Corresponding author: lguoqi1@lsu.edu (Dr. G. Li)

**Figure S1**. Typical plot of the thickness of the UV-cured 3D-RSMP resin as a function of the UV source input energy.


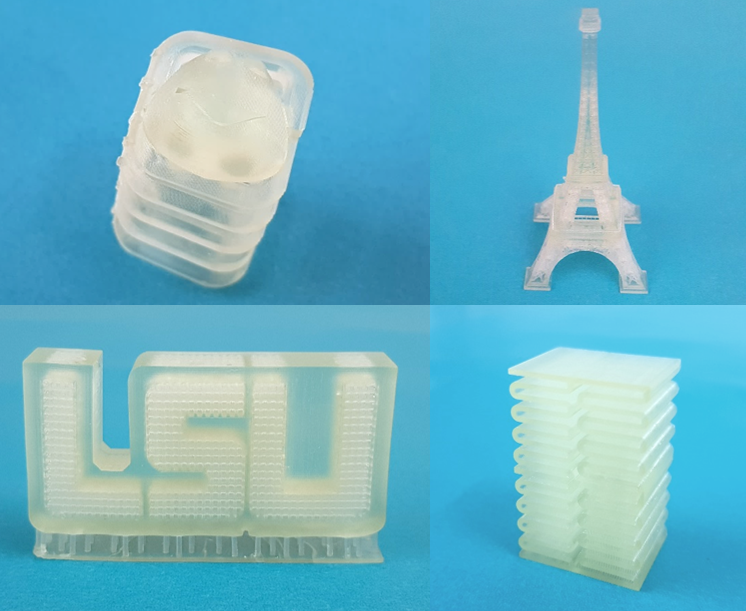


**(a)**


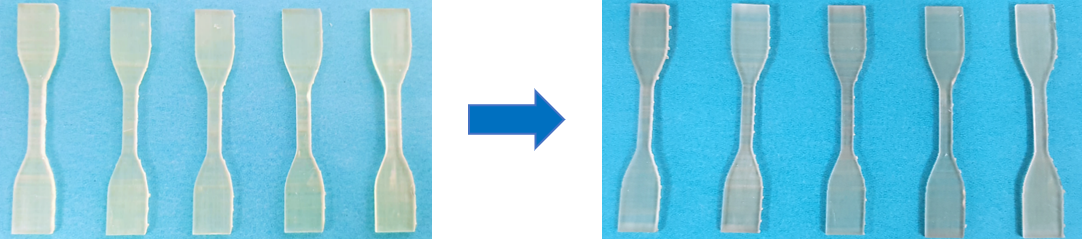


**(b)**


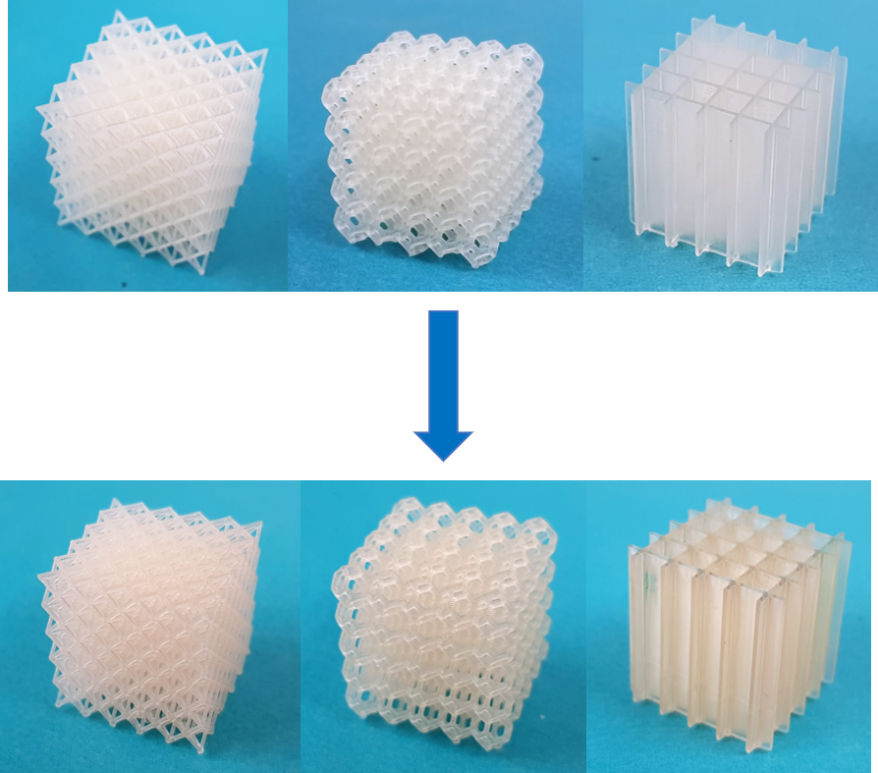


**(c)**

**Fig. S2.** **3D printed structures using the 3D-RSMP resin**. (**a)** a spring (upper left), an Effle Tower (upper right), a lattice LSU logo within a solid frame (lower left), a U-spring (2 × 2) structure (lower right). (**b)** 3D printed dogbone specimens color change from yellow immediately after printing to orange after 1 h post UV-curing (7.7 mW/cm^2^). (**c)** The color of the microlattices changed from pale yellow to orange after 1 h post UV-curing.

**Fig. S3.** **FTIR compositional analysis**. 3D-RSMP resin (black line) and the 3D printed 3D-RSMP powder made by ball milling (blue line).


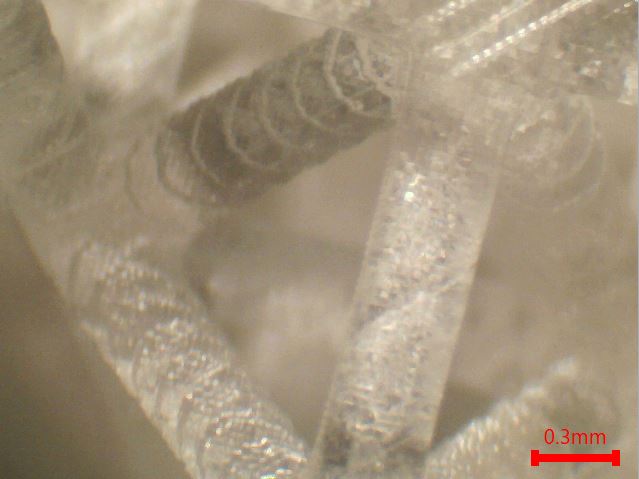

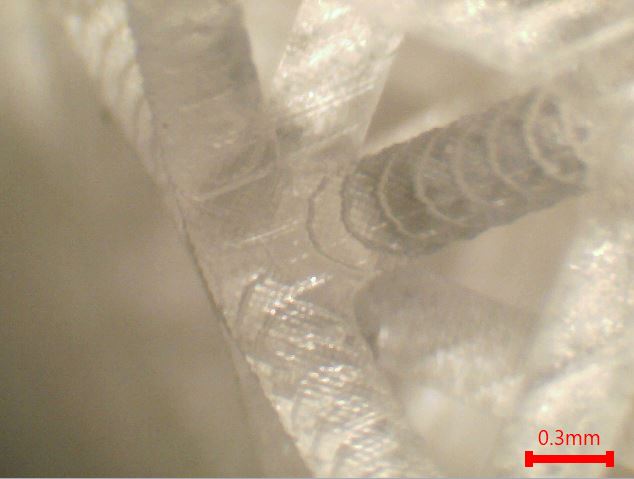


(**a) (b)**


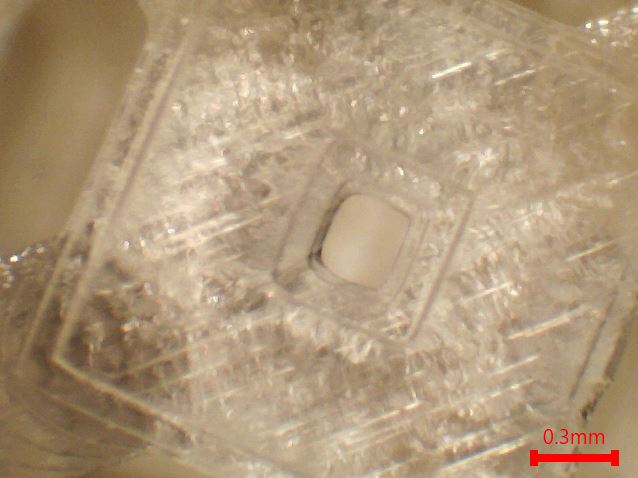

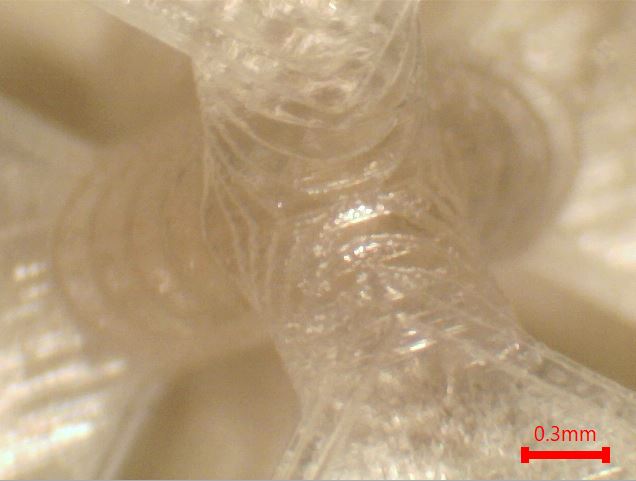


**(c) (d)**


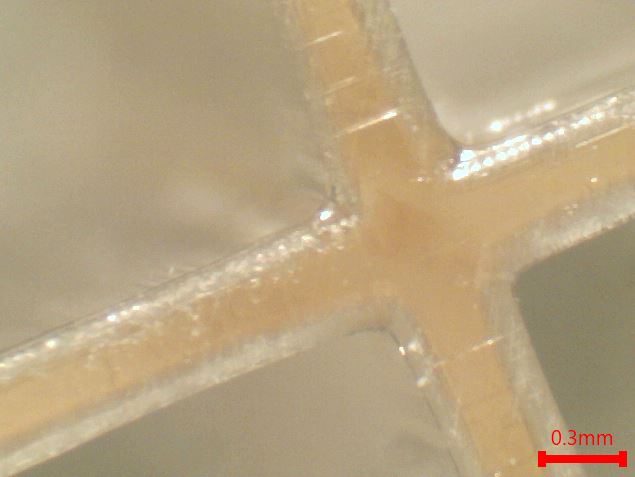

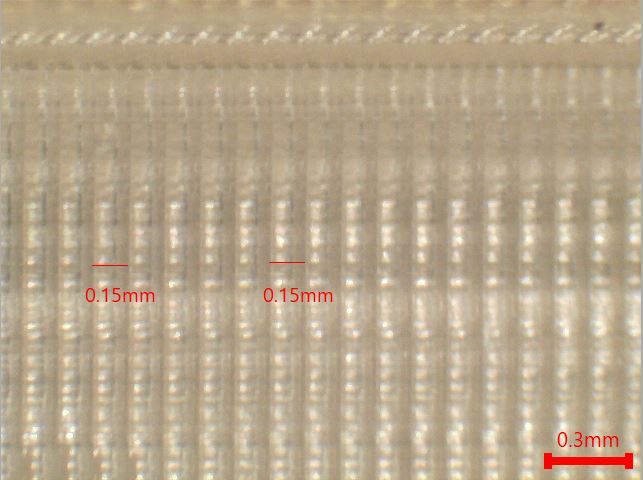


**(e) (f)**

**Fig. S4.** **Optical microscopic images of the printed microlattices with the 3D-RSMP resin.** (**a)** a horizontal strut of an octet microlattice, (**b)** inclined struts of an octet microlattice, (**c)** a flat square surface of a Kelvin microlattice, (**d)** an inclined strut of a Kelvin microlattice, (**e)** a flat surface of a cubic microlattice, and (**f)** a wall of a cubic microlattice.

**Fig. S5.** **Dynamic mechanical analysis.** Thermomechanical analysis of the 3D-RSMP with a temperature scan rate at 3 ^o^C/min.

**(a)**


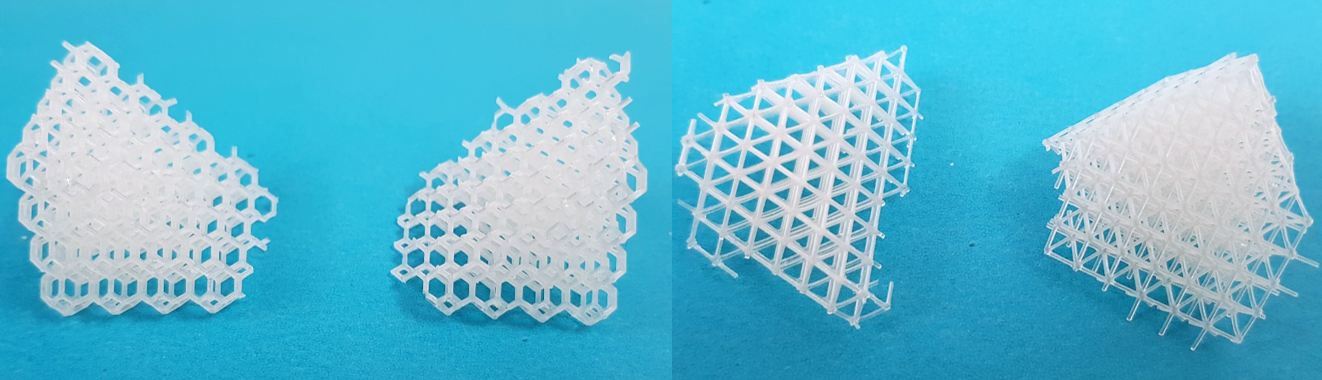


**(b)**

**Fig. S6.** **Compression behavior of microlattices.** (**a)** Typical compressive stress vs. strain curves of various cubic lattice structures (the numbers in the legend represent the apparent density in g/cm^3^). **b)** Collapsed Kelvin microlattices (left) and octet microlattices (right).

**Table S1.** **Representative values of compressive strength and modulus**

**of various microlattices**

| Items | Density  (g/cm^3^) | Relative density (ρ_A_/ρ_0_) | Compressive Strength (MPa) | Compressive Modulus (MPa) |
| --- | --- | --- | --- | --- |
| **Cubic** | 0.175 | 0.146 | 6.69 | 121.1 |
| **Microlattice** | 0.206 | 0.171 | 9.18 | 154.7 |
|  | 0.220 | 0.183 | 12.12 | 173.8 |
|  | 0.320 | 0.267 | 23.14 | 219.0 |
|  | 0.390 | 0.325 | 31.20 | 240.6 |
|  | 0.460 | 0.383 | 38.60 | 274.2 |
| **Octet** | 0.103 | 0.086 | 0.72 | 17.3 |
| **Microlattice** | 0.152 | 0.127 | 1.13 | 21.7 |
|  | 0.215 | 0.179 | 1.49 | 25.0 |
|  | 0.2765 | 0.230 | 2.55 | 35.8 |
|  | 0.310 | 0.258 | 3.85 | 76.1 |
|  | 0.400 | 0.333 | 5.80 | 91.6 |
|  | 0.520 | 0.433 | 6.55 | 108.5 |
| **Kelvin** | 0.103 | 0.086 | 0.26 | 9.6 |
| **Microlattice** | 0.188 | 0.157 | 0.61 | 42.5 |
|  | 0.253 | 0.211 | 2.50 | 50.0 |
|  | 0.324 | 0.270 | 2.76 | 77.9 |
|  | 0.390 | 0.325 | 3.13 | 145.4 |
| **1^st^ Order** | 0.127 | 0.106 | 1.39 | 34.4 |
| **Octet** | 0.183 | 0.153 | 1.45 | 52.2 |
| **Microlattice** | 0.228 | 0.190 | 1.51 | 75.0 |
|  | 0.306 | 0.255 | 2.40 | 102.0 |
|  | 0.395 | 0.329 | 5.10 | 129.0 |
| **2^nd^ Order** | 0.080 | 0.067 | 0.25 | 8.1 |
| **Octet** | 0.119 | 0.099 | 0.51 | 14.3 |
| **Microlattice** | 0.184 | 0.153 | 1.51 | 31.5 |
|  | 0.248 | 0.207 | 1.62 | 59.0 |
|  | 0.280 | 0.230 | 2.39 | 62.3 |
|  | 0.313 | 0.261 | 2.85 | 86.3 |

**(a)**

**(b)**

**Fig. S7.** **Mechanical properties of various microlattices or foams upon compression.** (**a)** Plots of compressive strength vs. apparent density (g/cm^3^) of various microlattices or foams in the logarithm scale. (**b)** Plots of relative modulus vs. relative density of various microlattices in the logarithm scale.

**(a) (b)**


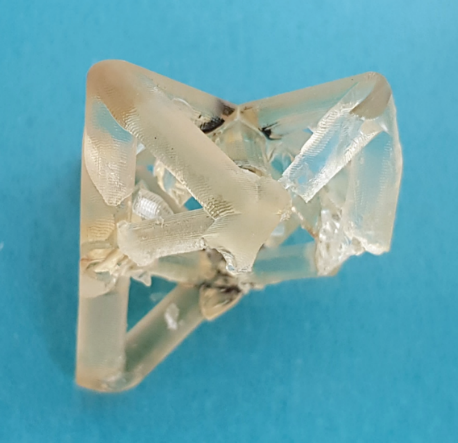

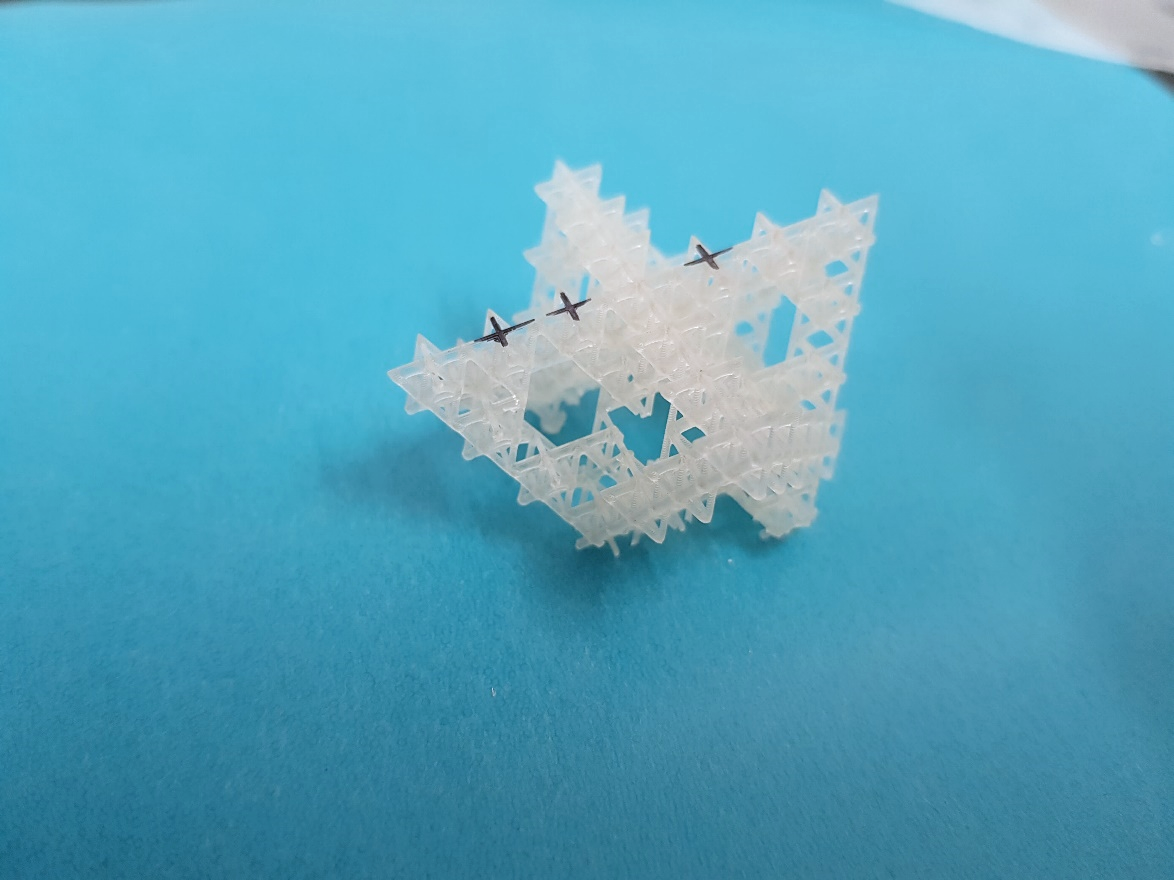


**(c)**

**Fig. S8.** **Mechanical properties of 1^st^ order octet microlattice and 2^nd^ order octet microlattice upon compression.** (**a)** Plots of compressive strength vs. apparent density. (**b)** Plots of compressive modulus vs. apparent density (g/cm^3^). **c)** Collapsed 1^st^ order octet microlattice (left) and 2^nd^ order microlattice (right).

**Table S2.** **Summary of mechanical properties and multifunctionality of the current 3D printable polymer and representative commercially available 3D printing resins**

| Category | Material | Tensile strength*^α^* (MPa) | Shape memory effect | Recovery stress (MPa) | Healed Tensile strength (MPa) |
| --- | --- | --- | --- | --- | --- |
| 3D/4D Printable vitrimer | Epoxy vitrimer *(46)* | 7 | N | N | around 6 |
|  | Thiole-ene photopolymer *(33)* | 0.12 | N | N | N/A |
|  | Urethane acrylate *(47)* | 5 | Y | N/A | around 0.7 |
|  | Acrylate thermoset *(54)* | 15*^β^* | N | N/A | Around 14 |
| 3D Printable shape memory polymers | PCL *(35)* | N/A | Y | N/A | N |
|  | Hybrid methacrylate photopolymers *(34* | 17.5 | Y | N/A | N/A |
|  | DGEBA Epoxy photopolymer *(49)* | 36.5 | Y | 4.7 | N |
| Commercial 3D printable ink | Veriguide-  *WhipMix* | 27.5 | N | N | N |
|  | 3DSR engineering hard resin-  *Kudo* | 34.9 | N | N | N |
|  | Tough resin-  *Formlabs* | 55.7 | N | N | N |
|  | VeroClear-  *Stratasys* | 65 | N | N | N |
| This study (3D-RSMP resin) | Multifunctional Epoxy Methacrylate | 62 | Y | 12.1 | 17.6 |

*^α^*Tensile strength of the 3D printed specimen.

*^β^*The mechanical results were obtained after 0- 4 h thermal treatment.
